# Exosomes derived from MSC as drug system in osteoarthritis therapy

**DOI:** 10.3389/fbioe.2024.1331218

**Published:** 2024-03-20

**Authors:** Shuzhan Wen, Xin Huang, Jingchun Ma, Guanglei Zhao, Tiancong Ma, Kangming Chen, Gangyong Huang, Jie Chen, Jingsheng Shi, Siqun Wang

**Affiliations:** Department of Orthopedics, Huashan Hospital, Fudan University, Shanghai, China

**Keywords:** exosome, mesenchymal stem cell, osteoarthritis, drug delivery, engineered exosome

## Abstract

Osteoarthritis (OA) is the most common degenerative disease of the joint with irreversible cartilage damage as the main pathological feature. With the development of regenerative medicine, mesenchymal stem cells (MSCs) have been found to have strong therapeutic potential. However, intraarticular MSCs injection therapy is limited by economic costs and ethics. Exosomes derived from MSC (MSC-Exos), as the important intercellular communication mode of MSCs, contain nucleic acid, proteins, lipids, microRNAs, and other biologically active substances. With excellent editability and specificity, MSC-Exos function as a targeted delivery system for OA treatment, modulating immunity, inhibiting apoptosis, and promoting regeneration. This article reviews the mechanism of action of MSC-Exos in the treatment of osteoarthritis, the current research status of the preparation of MSC-Exos and its application of drug delivery in OA therapy.

## 1 Introduction

Osteoarthritis (OA) is one of the most prevalent forms of arthritis. Data from 2021 indicate that 22% of adults aged 40 and above suffer from knee OA. It is estimated that over 500 million individuals worldwide are affected by OA, making it the fourth leading cause of disability (Quicke et al., 2022). The substantial burden it places on healthcare resources, coupled with the loss of productivity due to unemployment and early retirement, imposes significant societal challenges. Osteoarthritis is a complex degenerative disease involving various components within the synovial joint. Its primary pathological features include the loss of articular cartilage, subchondral bone remodeling, inflammatory changes in synovial tissues, and instability of tendons and ligaments (Hunter and Bierma-Zeinstra, 2019). Among these, the irreversible damage to cartilage has long presented a therapeutic challenge.

Due to the avascular nature of cartilage tissue, its capacity for self-repair is severely limited. Mature chondrocytes have limited proliferative abilities, and the synthesis and metabolism of surrounding cartilage cells alone are insufficient to fill cartilage defects (Wakitani et al., 2004). Currently, there are few drugs available for the clinical treatment of cartilage injuries. Oral medications struggle to reach effective concentrations within joint cavities, and even intra-articular injections are quickly absorbed by lymphatic fluid, resulting in brief therapeutic effects and a heightened risk of infection (Patel et al., 2019). Therefore, research into more potent targeted therapies and effective drug delivery systems is of paramount importance.

In recent years, researchers have continuously developed novel biologics such as growth factors, anti-inflammatory agents, and Platelet-rich plasma (PRP) in an attempt to slow down the progression of OA by nourishing cartilage and suppressing intra-articular inflammatory responses (Hickey et al., 2003; Liang et al., 2022). Mesenchymal stem cells (MSCs) are a type of multipotent stem cell known for their self-renewal and multi-lineage differentiation capabilities. They can influence biological processes through paracrine effects, exhibiting anti-inflammatory, analgesic, and immune-modulating properties, have demonstrated promising results in cartilage repair research (Le et al., 2020).

Exosomes derived from MSCs (MSC-Exos) contain various proteins, mRNAs, and miRNAs, playing crucial roles in intercellular communication. Compared to MSCs, MSC-Exos offer advantages such as enhanced safety, ease of storage, minimal side effects, and fewer ethical concerns (Mendt et al., 2019). Advances in biotechnological techniques further harness their potential in drug delivery systems. This review focuses on the clinical prospects of MSC-Exos in OA treatment. It summarizes the biological characteristics, preparation methods, and explores their potential as a drug delivery system for OA therapy.

## 2 MSC and MSC-Exos in OA progression

Chondrocytes are the primary cellular components of articular cartilage, enclosed within a dense extracellular matrix (ECM). Under normal conditions, these cells maintain a delicate balance between ECM synthesis and degradation (Ruhlen and Marberry, 2014). However, this equilibrium can be disrupted by factors such as aging, metabolic changes, and mechanical stress, leading to a compromise in cartilage’s structural and functional integrity (Di Rosa et al., 2014). In the early stages of OA, chondrocytes function as environmental sensors within the joint, but their natural repair capacity is limited. Efforts to sustain ECM composition often involve transiently boosting ECM synthesis through pathological changes, such as chondrocyte hypertrophy, marked by increased expression of indicators like Runx2, MMP13, and CoIX. Consequently, these alterations stimulate the production of factors contributing to cartilage degeneration (Xu et al., 2014). While a small fraction of MSCs within the joint cavity plays a role in cartilage repair, their impact is relatively modest. Research has indicated that MSCs or synovial cells present in synovial fluid can be mobilized to the injury site under the influence of growth factors, aiding in cartilage defect repair. However, their effectiveness remains limited (Sekiya et al., 2012).

As the disease progresses, chondrocyte activity becomes dysregulated, hindering proliferation and synthetic metabolism. Cartilage defects worsen, extending to the subchondral bone. In this stage, local blood and MSCs infiltrate the area, forming a hematoma comprising fibrin clots, along with platelet, red blood cell, and white blood cell accumulations. MSCs, primarily derived from bone marrow, secrete various cytokines, including insulin-like growth factor 1, transforming growth factor-β, and platelet-derived growth factor, participating in the repair process (Hickey et al., 2003). Within 2 weeks of injury, MSCs transform into chondrocyte-like cells, synthesizing and secreting new ECM rich in type II collagen and proteoglycans. This tissue, resembling hyaline cartilage, fills the defect. Six to 8 weeks later, this newly formed tissue may transition from fibrous cartilage to hyaline cartilage. However, even if the defect is successfully filled, the resulting cartilage exhibits reduced hardness and low resistance to compression, making it vulnerable to further damage under mechanical loads. In the long term, neighboring chondrocytes may also be impacted, increasing the risk of osteoarthritis spreading beyond the initial localized area (Mobasheri et al., 2002).

OA is no longer regarded as a simple degenerative disease but rather a multifaceted condition involving a interplay of local and systemic factors (Hunter and Bierma-Zeinstra, 2019). The pathology extends across the entire joint, encompassing not only cartilage damage but also synovial membrane thickening, subchondral bone sclerosis, osteophyte formation, and structural alterations in ligaments and surrounding muscles. In the process of OA progression, the interactions between different tissues within the joints are crucial mechanisms in the pathophysiology of OA (Sharma et al., 2013). Mechanical stress between bone and cartilage, as well as synovial inflammation, affects cartilage degeneration (de Lange-Brokaar et al., 2012). Conversely, the altered cartilage cells impact other components within the joint. Extracellular vesicles (EVs) are recognized as vital mediators of intercellular communication. Their role in OA progression within the joints has gained increasing attention. Exosomes released by chondrocytes have a dual effect on OA. EVs secreted by healthy chondrocytes can regulate M2 macrophage penetration, facilitating the elimination of mitochondrial dysfunction and promoting immune recovery (Sun et al., 2019). However, EVs from OA chondrocytes promote autophagy mediated by miR-449a-5p/ATG4B, inhibiting chondrocyte proliferation, enhancing chondrocyte apoptosis, and upregulating the production of pro-inflammatory mediators by macrophages (Ni et al., 2019).

Numerous studies have demonstrated that exosomes derived from mesenchymal stem cells (MSCs) can slow down the progression of OA. Many scholars have proven that MSC-based OA therapy primarily operates through EVs, delivering substances including small RNAs. These substances regulate various biological activities such as chondrocyte apoptosis and senescence, synovial cell proliferation, and the release of pro-inflammatory mediators (Cosenza et al., 2017). EVs exhibit specific cell targeting properties based on their characteristics and sources, and they are biocompatible (Batrakova and Kim, 2015). Therefore, extensive research efforts have been made to delay OA development using MSC-Exos. Ongoing advancements in engineering techniques aim to develop drug delivery systems based on MSC-Exos, paving the way for new approaches in OA treatment.

## 3 Biological characteristics of MSC-Exos

### 3.1 Architecture of exosomes

Exosomes, with a diameter of about 40–100 nm, constitute a subset of biological nano-scale spherical lipid bilayer vesicles that have been found to be secreted by most cell types. The term “exosomes” was initially coined in 1981 by Trams et al. (1981), referring to vesicles derived from the plasma membrane. These vesicles were proposed to possess 5′-nucleotide enzyme activity, potentially serving physiological functions and originating from the extrusion of diverse cell line cultures (Harding et al., 1983). Distinguishing exosomes from other extracellular vesicles (EVs), such as microvesicles (MVs) and apoptotic bodies (ABs), can be challenging due to inherent heterogeneity both between and within exosome subtypes, along with overlapping characteristics. Notably, exosomes have been found to encapsulate a rich array of bioactive substances, including proteins, nucleic acids, lipids, cytokines, transcription factor receptors, and more (Théry et al., 2002). The composition of exosomal plasma membranes comprises a variety of lipids, encompassing hexosylceramides, cholesterol, phosphatidylserine, sphingomyelin, and saturated fatty acids (Skotland et al., 2019). Exosomal protein constituents can be broadly classified into two categories: common components, integral to vesicle formation and secretion, and specific components, intimately linked to the cell of origin. Common components encompass a range of proteins that play pivotal roles in processes such as membrane transport and fusion (e.g., Rab GTPases), heat shock responses (e.g.,,HSP70, HSP90), proteins belonging to the four-transmembrane protein superfamily (e.g.,,CD63, CD81), endosomal sorting complex required for transport (ESCRT)-related proteins (e.g., Tsg101, Alix), and integrins, etc. The other one is specific components, which are closely related to their progenitor cells including cell-specific markers like CD45 and MHC-II, which originate from antigen-presenting cells (Henne et al., 2011; Juan and Fürthauer, 2018).

### 3.2 Biogenesis of MSC-Exos

EVs biogenesis starts within the cell endosomal system. Unlike MVs and ABs, exosomes are not released directly from the plasma membrane, but are generated by inward budding of the multivesicular bodies (MVBs) membrane. MVBs referred to as late sorted endosomes (LEs), invagination of endosomal membranes occures during the maturation of early endsomes into LEs, results in the formation of intraluminal vesicles (ILVs) (Minciacchi et al., 2015). This process causes some cytoplasmic components to be engulfed and enclosed in ILVs, most of them are released outside the cell through fusion of MVBs with plasma membrane and part of them are transported to lysosomes for degradation. Those ILVs released into extracellular space are referred to as “exosomes” (Sahu et al., 2011). In 2010, Sai Kiang Lim’s team first demonstrated the therapeutic effects of MSCs through the secretion of exosomes (Lai et al., 2010). Subsequently, they confirmed that MSC-Exos also originate from endosomes and are rich in GM1 gangliosides, exhibiting high internalization activity (Tan et al., 2013). Further studies have consistently shown that MSC-Exos share similar biomarkers with exosomes derived from other cell sources like Alix, Tsg101, CD9, CD63 or CD81 (Kowal et al., 2016).

ESCRT consists of five functional subcomplexes (ESRCT-0,-I,-II,-III and AAA ATPase Vps4) were sequentially discovered to regulate the budding and release ILVs. Some studies suggest that ILVs can occur in MVBs without ESRCT, the biogenesis of exosomes can now be categorized into ESCRT-dependent and ESCRT-independent mechanisms (Henne et al., 2011). The role of the RAB family of small GTPase proteins, including Rab27a, Rab27b, Rab35, and Rab7, in facilitating vesicular transport between intracellular compartments is well-established. These proteins have also been implicated in orchestrating the intracellular trafficking of vesicles to the plasma membrane, ultimately leading to the release of exosomes. Additionally, the biogenesis of MSC-Exos also depend on extracellular condition. For instance, exosomes derived from MSCs stimulated by hypoxia condition exhibit a higher level of angiogenic activity.

### 3.3 Biological advantages of MSC-Exos as drug carrier

EVs derived from human cells possess remarkable biocompatibility, low toxicity, and minimal immunogenicity, especially when obtained from autologous sources. MSC-Exos, in particular, have gained significant attention in the treatment of various conditions, including osteoarthritis, tumors, and autoimmune diseases (such as IBD, SLE, and SS), with rare significant adverse reactions reported (Wiklander et al., 2015; Fang et al., 2023). Their bilayered lipid vesicle structure and nanoscale dimensions enable them to remain stable during long-distance transport and penetrate tissue barriers, including the joint cavity. They have demonstrated effective traversal of biological barriers like the blood-brain barrier (BBB) and placental barrier (Rehman et al., 2023). EV markers such as CD55 and CD59 prevent complement and coagulation factor activation, ensuring their widespread and stable distribution in body fluids.

The diverse contents within EVs, including proteins, lipids, and various nucleic acids (such as miRNAs, mRNAs, tRNAs, lncRNAs, and rRNAs), exhibit excellent biocompatibility with various drugs. Besides therapy, they can also carry contrast agents for disease diagnosis (Avgoulas et al., 2023). In many cases, EV-mediated delivery constitutes the primary mechanism of cell therapy. One significant advantage of drug-loaded EVs is their ability to retain certain characteristics of the parent cells. Meanwhile, the characteristics and applications of MSC-Exo from different sources also vary (Table 1). For instance, immune cell-secreted EVs possess robust immunomodulatory capabilities, and studies indicate that mesenchymal stem cell-derived EVs exhibit both immunomodulatory and regenerative functions. MSC-Exos can reduce the expression of inflammatory mediators through various pathways. For example, Zhang et al. demonstrated that MSC-Exos could attract M2 macrophages, reducing the infiltration of pro-inflammatory M1 macrophages and downregulating the expression of cytokines like IL-1β and TNF-α, thus inhibiting inflammation in osteoarthritis (Zhang et al., 2018b). Moreover, when co-cultured with chondrocytes, adipose-derived mesenchymal stem cells (AD-MSCs) and chondrocytes interact through EVs, promoting AD-MSCs chondrogenesis, increasing chondrocyte extracellular matrix synthesis, and inhibiting cell senescence.

**TABLE 1:** Comparison of several commonly used MSC-Exos in drug delivery.

Sources	Tissue acquisition	Surface marker	Advantages	Disadvantages	Application	References
BMSCs	Biopsy/Aspiration from posterior superior iliac pole or sternum, Bone Marrow Banks	CD9 CD44	Exosomes yield low cost	Invasive collection surgery. Limited donors	Bone diseases, Arthritis, Fracture healing, Cardiovascular diseases, Autoimmune diseases, etc.	Liu et al. (2022)
		CD61CD81	High proliferative capacity	Limited efficiency of exosome production		Shao et al. (2022)
			High potential to keep chondrocytes viability			Jiang et al. (2020)
AD-MSCs	Liposuction (commomly removed from abdomen thighs or buttocks), Fat Grafts in surgeries (like abdominoplasty or breast reduction), SVF isolation	CD9 CD63 CD29 CD81 Alix TSG101	Rich source and easy to obtain	Invasive adipose tissue collection, lower exosome content	Cosmetic surgery, Soft tissue repair, Arthritis, Organ transplant, Diabetes, etc.	Smith et al. (2021)
			High efficacy of exosome production and can be enhanced by preconditi-oning media			Cai et al. (2020)
UC-MSCs	Umbilical cord blood collected from the umbilical vein at the time of childbirth, Wharton’s Jelly Extraction	CD9 CD61 CD8 Alix TSG101	Non-invasive acquisition from umbilical cord waste	Limited collection time. Strict storage conditions	Neurological disorders, Cardiovascular diseases, Liver diseases, Lung diseases, Autoimmune diseases, etc.	Zhao et al. (2019)
			Show more ability for hepatocytes proliferation	Ethical limitations		Yaghoubi et al. (2019)
PL-MSCs	Placental tissue obtained after childbirth, Cryopreserved Placental Tissue	ULBPs CD9 CD81 CD61 CD105 NKG2D	Non-invasive acquisition from placenta waste	Donor restrictions	Fetal developmental disorders, Autoimmune diseases, Diabetes, Cardiovascular diseases, etc.	Komaki et al. (2017)
			High capacity of renewal	Strict storage conditions		Hass et al. (2011)
				Ethical limitations		
SF-MSCs/SMSCs	Biopsy during arthro-scopy, Arthrocentesis	CD44 CD73 CD90	High differentiation and proliferation capacity	Limited tissue	Bone and joint diseases, Sports injuries, etc.	To et al. (2019)
			Significant effect in joint-related diseases	Lower exosome content		Chen et al. (2018)
				More impurities		Zhu et al. (2017)

Abbreviations: UC-MSCs, Umbilical cord mesenchymal stem cells; PL-MSCs, Placenta-derived mesenchymal stem cells; SF-MSCs, Synovial fluid-derived mesenchymal stem cells; SMSCs, Synovial mesenchymal stem cells.

MSC-Exos offer significant advantages in terms of targeting ability and editability, greatly enhancing their drug delivery efficiency. After release from parent cells, exosomes can be internalized by targeted recipient cells through various endocytic pathways, including clathrin-dependent and clathrin-independent endocytosis, phagocytosis, and micropinocytosis. If not internalized, they can attach to target cell membranes through ligand-receptor interactions, activating downstream signaling cascades. For instance, the specific ligands on the surface of AD-MSCs-Exos allow them to target chondrocytes. When loaded with miR-29a and miR-29b, these MSC-Exos can directly target the 3’untranslated region of the Collagen-1 gene, influencing cartilage development and homeostasis (Yan et al., 2011). MSC-Exos exhibit strong editability; alterations in parent cell selection and modifications in culture conditions can enhance their bioactive composition. Techniques such as surface modifications and genetic modifications have significantly improved their drug loading efficiency and targeting capabilities, as will be discussed in detail later in this article.

**FIGURE 1:**
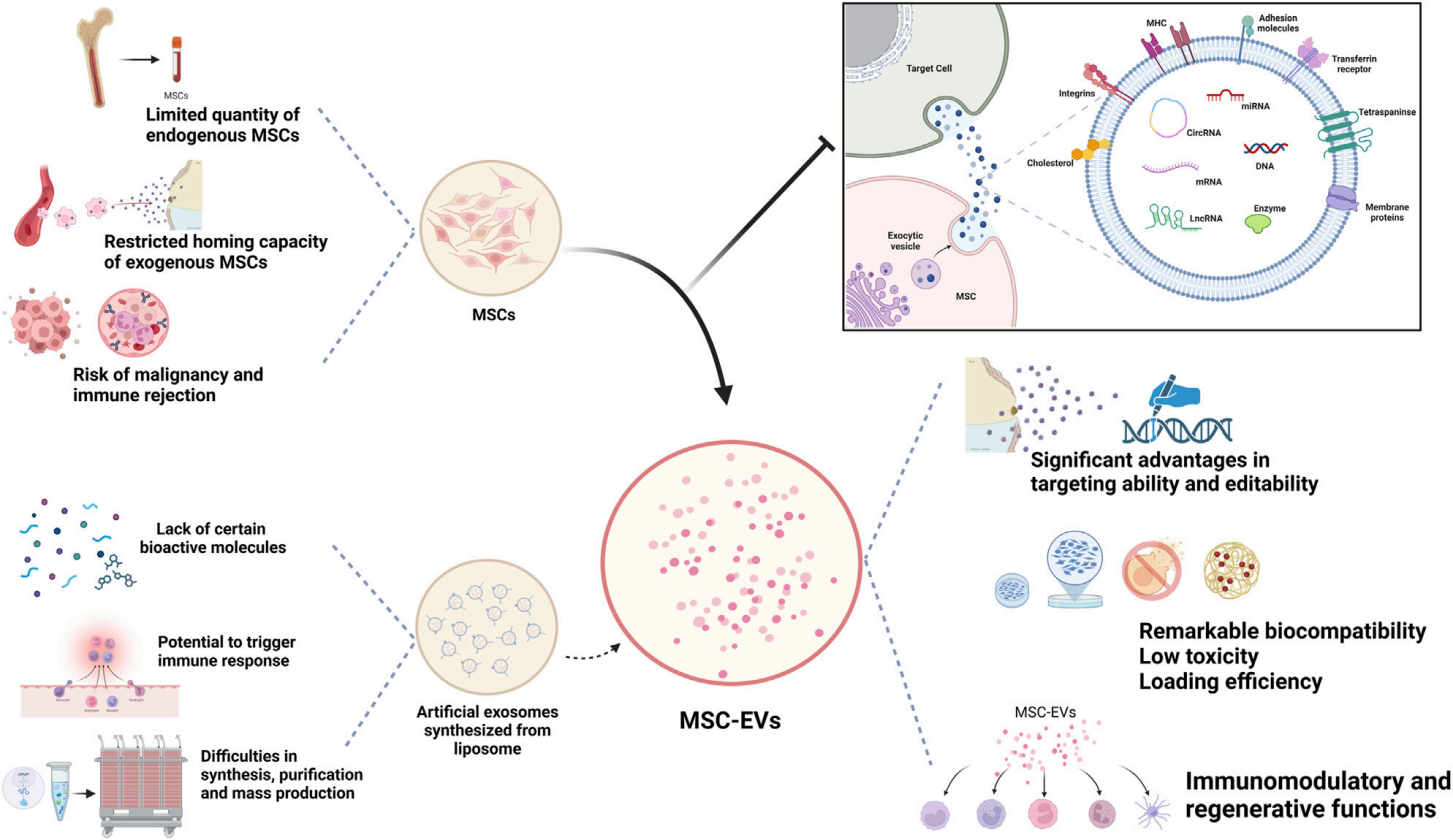
Biological advantages of MSC-Exos as drug carrier.

Unlike cell therapy, EVs face fewer ethical restrictions and a lower risk of immune rejection and malignancy. Meanwhile, compared with MSCs, which require strict conditions to maintain their vitality, MSC-Exos can be easily maintained and stored (Okamura et al., 2023). Currently, many researchers utilize artificially derived extracellular vesicles, such as lipid nanoparticles and liposomes, for their studies because they allow for precise engineering design and offer higher reproducibility. However, they lack the inherent biological complexity of MSC-EVs, often carrying single bioactive molecules, and cannot fully replicate the biological functions of MSC-Exos (Li et al., 2021). Therefore, naturally sourced EVs offer superior therapeutic applicability compared to synthetic drug delivery systems like nanoparticles, liposomes, and resin polymers (Figure 1).

## 4 Preparation of MSC-Exos

### 4.1 Sources and preparation of MSC

MSCs can be sourced from various tissues, including the umbilical cord, adipose tissue, muscle, dental tissue, salivary glands, menstrual blood, and more. Despite their diverse origins, they share common traits such as self-renewal, multi-lineage differentiation potential, and immunomodulatory properties. The International Society for Cellular Therapy has defined three basic criteria for identifying human MSCs: (1) When maintained under standard culture conditions, MSCs must adhere to plastic surfaces. (2) MSCs must express CD105, CD73 and CD90, while not expressing CD45, CD34, CD14, CD11b, CD79a, CD19 or HLA-DR surface markers. (3) MSCs must be capable of differentiating into osteoblasts, adipocytes, and chondrocytes *in vitro* (Dominici et al., 2006). MSCs can be isolated from various joint tissues, including synovial tissue, meniscus, ligaments, adipose pads, and cartilage. MSCs can be isolated from various joint tissues, including synovial tissue, meniscus, ligaments, adipose pads, and cartilage.

The exosomes derived from bone marrow mesenchymal stem cells (BMSCs) were initially used in OA treatment due to their easy isolation, proliferation, and storage in clinical settings. However, BMSCs face challenges such as decreased stem cell number and differentiation capacity with aging, traumatic extraction leading to higher pain levels and complications, and limited extractable bone marrow quantity (Li and Zhang, 2019). In contrast, AD-MSCs have a broader source distribution, as adipose tissue is widely found in the body. By processing adipose tissue through techniques like centrifugation and filtering, the stromal vascular fraction (SVF) can be obtained, containing various cells and cytokines, including mesenchymal stem cells. AD-MSCs can be extracted from SVF, mainly obtained through liposuction procedures. While traditional techniques liposuction yield a high amount of fat, there are still risks of damage to soft tissues, blood vessels, nerves, postoperative uneven skin surfaces, and subcutaneous hematomas (Bourin et al., 2013). Upgraded techniques such as hydrodynamic/ultrasound/laser-assisted negative pressure suction reduce complications and yield more active AD-MSCs. Nowadays, researchers are exploring the extraction of adipose tissue from other surgical procedures, such as subcutaneous fat pads removed during knee arthroscopy. Human adipose tissue is abundant, the extraction process is simple, less ethically constrained, and adipose tissue contains a high proportion of stem cells in the stromal fraction, approximately 5%, whereas BMSCs only account for 0.0001%–0.01%, allows for faster expansion and proliferation (Eom et al., 2011).

Standardized preparation procedures for most MSCs sources, including tissue mincing, optional enzymatic digestion, and explant culture, are lacking. For instance, the cultivation of mature AD-MSCs typically involves the following steps: first, cleaning the obtained adipose tissue with PBS to remove excess blood components, local anesthetics, or joint effusion. Next, the tissue is finely chopped, and collagenase is utilized to eliminate fibrillar collagen. After centrifugation and filtration, the separated fat scraps are introduced into the culture medium for cell growth. These cells are usually passaged to the 3rd to 5th generation for subsequent experiments. There are variations in basic culture media, types and concentrations of collagenase, digestion time, centrifugation speed, duration, and other details among different laboratories (Eydt et al., 2016). Some researchers have attempted to enhance AD-MSCs digestion efficiency using matrix gel digestion methods. However, AD-MSCs consistently yield higher quantities compared to BMSCs (Gittel et al., 2013).

The identification of MSCs involves flow cytometric analysis of their surface markers. Surface marker profiles differ among MSCs from various sources. Both AD-MSCs and BMSCs express CD13, CD29, CD34, CD44, CD73, CD90, CD105, CD166, MHC class I, and HLA-ABC on their cell surfaces. Notably, AD-MSCs do not express CD38, CD45, CD106, HLA-DR, DP, DQ (MHC class II), CD80, CD86, CD40, or CD154 (Zimmerlin et al., 2010). The activity of AD-MSCs can be influenced by patients, sites of adipose tissue, and the culture media used. Common methods for assessing AD-MSCs activity include alkaline phosphatase, Safranin O, Masson’s trichrome staining, ELISA, and Western blot experiments. Researchers, such as Schipper et al., have studied AD-MSCs from adipose tissue obtained from various age groups of women and discovered that the activity of AD-MSCs is significantly higher in younger women. Additionally, adipose tissue from the superficial abdominal layer offers advantages over tissue from the upper arm, inner thigh, rotator cuff, and deep abdominal tissue. In male patients, abdominal superficial tissue also demonstrates advantages over deep fat tissue (Mukhamedshina et al., 2019).

### 4.2 Isolation and identification of MSC-Exos

As a drug carrier within the body, efficiently obtaining pure, safe, and sufficient exosomes from MSCs is of paramount importance. Ensuring the purity and homogeneity of exosomes while achieving high-efficiency large-scale production has posed technical challenges. Several methods have been employed for isolating exosomes from MSCs, with differential centrifugation being the most traditional approach. Currently, MSCs from the 3rd to 6th generation are generally used. When cell confluence reaches 80%–90%, serum-free medium is replaced, and cells are cultured for 24–48 h (Jha et al., 2016). The resulting cell culture supernatant contains not only exosomes but also various components like dead cells, cell fragments, microvesicles, and apoptotic bodies. Ultracentrifugation is employed to separate exosomes based on size and density, followed by identification using transmission electron microscopy (TEM). Differential centrifugation yields high purity but is less efficient. In contrast, ultrafiltration is more suitable for large-scale production due to lower equipment requirements and shorter processing times. Size Exclusion Chromatography (SEC) and Ion Exchange Chromatography (IEC) also yield high-purity exosomes (Koh et al., 2018). Immunoisolation, based on the specific binding of antibodies targeting surface proteins like CD9, CD63, CD81, Alix, and heat shock proteins, offers high purity but is less suitable for large-scale production. However, its high specificity can be used to isolate specific subpopulations of exosomes, holding unique clinical value (Doyle and Wang, 2019).

Exosome identification techniques have matured, with no significant differences in morphology, isolation, and storage conditions between exosomes derived from MSCs and those from other sources. The basic steps for exosome identification involve morphological observation and surface marker identification. Transmission electron microscopy (TEM) and scanning electron microscopy (SEM) are commonly used techniques for exosome observation (Gurung et al., 2021). Cryo-electron microscopy (cryo-EM) provides more accurate biological information, offering higher resolution and allowing imaging of exosomes in their natural hydrated state without chemical fixation or staining, preserving their natural structure. Cryo-electron tomography (Cryo-ET), an extension of cryo-EM, provides detailed structural information through three-dimensional imaging of exosomes.

Exosomes derived from MSCs not only express common surface markers such as CD9 and CD81 but also often express adhesion molecules like CD29, CD44, and CD73 found on MSC membranes. Developing more sensitive and specific surface protein detection methods has been a focus of exosome identification research. Advanced mass spectrometry techniques enable comprehensive analysis of exosomal protein content and help identify potential biomarkers associated with specific diseases or cellular functions. Immunomagnetic bead-based methods and flow cytometry are also commonly used identification methods. High-throughput sequencing has been used to identify and analyze various RNA and protein contents in exosomes. Nanotechnology and microfluidics techniques allow the analysis of individual exosomes. Diogo Fortunato et al. used nanopore sensing and super-resolution microscopy to identify and analyze exosomes from different cell types, providing a valuable method for exosome heterogeneity analysis (Fortunato et al., 2021).

## 5 MSC-Exos engineering for drug delivery

### 5.1 Methods to load cargo into MSC-Exos

One of the fundamental challenges in utilizing MSC-Exos for drug delivery lies in efficiently loading them with various therapeutic cargo. There are two primary strategies. In one approach, therapeutic drugs are loaded into parent MSCs, where they are encapsulated within exosomes during their formation. Nucleic acid cargo is often introduced into cells through viruses or plasmid vectors, enriching therapeutic agents in parent cells to secrete engineered exosomes. To facilitate the entry of target proteins into exosomes, researchers merge the target proteins with anchoring proteins known to enter exosomes. Alternatively, modifications such as WW tag and ubiquitination are employed (Sterzenbach et al., 2017). WW tag, consisting of 38–40 amino acid residues forming a three-strand β-fold structural domain, can be recognized by the late structural domain base sequence on Ndfipl, promoting the packaging of target proteins into exosomes (Lu et al., 1999). Small-molecule chemical drugs are often directly loaded into purified exosomes, necessitating overcoming the barrier function of the exosomal membrane. Hydrophobic membrane-permeable molecules can be delivered into exosomes by simple coincubation at certain temperature and pH. Electroporation is a common method for the passive loading of exogenous cargo. It involves temporarily creating small pores in the phospholipid bilayer using an applied electric field. Under the influence of the electric field, drugs enter the exosomes and then the integrity of the exosomal membrane is restored. Similarly, sonication employs soundwaves to temporarily destabilize the exosomal membrane, facilitating cargo incorporation (Wan et al., 2022). Colja et al. (2023) utilized exosomes to deliver the anti-vimentin nanobody Nb79 for treating Glioblastoma, they experimented with four different methods to load the antibody into the exosomes. The final results indicated that sonication proved to be an efficient method for cargo loading.

### 5.2 Technologies of large-scale exosome production

To meet the demands of exosome-based drug delivery, researchers have explored diverse strategies aimed at increasing exosome yield. One such approach involves the overexpression or modification of key genes related to processes vital for exosome biogenesis, such as membrane binding and MVBs transportation. For instance, enhancing the expression of TSPAN6 and tetraspanin CD9 has been found to release more exosomes by interacting with multifunctional cytosolic adaptors (Schiller et al., 2018). Another avenue explored by researchers is the genetic modification of MSCs, where the introduction of the MYC gene results in significantly elevated MSC-Exo production. However, the use of MYC, being an oncogene, raises apprehensions regarding its clinical safety (Li et al., 2022).

In addition to biochemical stimuli, researchers have endeavored to optimize cell culture equipment. Utilizing innovative methods such as scaffolds, spheroid culture, hollow fiber bioreactors, and microcarrier-based 3D culture techniques, scientists aim to maximize the limited surface area available for cell growth. Interestingly, expanding the cell-to-cell contact area not only facilitates exosome secretion but also promotes cell differentiation and enhances immunoregulatory capabilities. According to reports, 3D spheroid cultures not only augment MSC exosome secretion but also strengthen their anti-inflammatory and pro-angiogenic functions, showcasing the multifaceted benefits of such innovative culture methods (Kim et al., 2018). Stirred tank bioreactors (STRs) achieve mass transfer and control of cultivation parameters, such as temperature, pH, dissolved oxygen, nutrients, and metabolites, through the circulation generated by mixing and aeration. The advantages of this system lie in its scalability, the uniformity it provides, and the controllability of cultivation variables. It can be adjusted to suit various cell types and specific purposes and is suitable for large-scale exosome production (Eibl et al., 2010).

### 5.3 Surface modification for targeted delivery

The targeted delivery of exosome derived from natural sources is limited, and even exosomes sourced from the same type of cells exhibit significant heterogeneity. Enhancing the targeting ability of exosome loaded with drugs is crucial for improving drug efficiency, reducing side effects, and directly impacting the overall therapeutic outcome. Exosomes sinternalize into cells by fusing with the cell membrane and activating specific signaling pathways through ligand-receptor interactions. Therefore, the most commonly adopted strategy is to modify the surface of exosomes through genetic or chemical methods to enhance their targeting ability. MSCs can undergo genetic modification to produce exosomes with specific surface proteins or peptides, enabling targeted delivery to chondrocytes or synovial cells in osteoarthritic lesions. Jiang Xia et al. fused chondrocyte-affinity peptide (CAP) with lysosome-associated membrane glycoprotein 2b protein on the surface of exosomes (Liang et al., 2020). This modification allowed the exosomes to effectively encapsulate miR-140 and specifically deliver the cargo to extracellular chondrocytes. Importantly, these CAP-modified exosomes were proven to be well retained within the joint cavity after intra-articular injection. Furthermore, they could be delivered to the deep cartilage regions, inhibiting cartilage-degrading proteases effectively (Tao et al., 2017).

MSC-Exos can also be combined with chemically engineered materials that possess targeted properties, such as temperature sensitivity, pH sensitivity, and charge affinity, to enhance their efficacy. For instance, Schiffelers et al. coated phospholipids (DMPE) and polyethylene glycol (PEG) on the membrane of exosomes containing EGFR nanoantibodies. The modified exosome membrane exhibited temperature-dependent fusion characteristics, significantly enhancing its specific binding to tumor cells overexpressing EGFR. After intravenous injection in mice, the modified exosomes could still be detected in the plasma after 60 min, whereas unmodified exosomes were rapidly cleared within 10 min (Meldolesi, 2018). As OA progresses, physiological factors within the joint cavity, such as ROS level, temperature, and pH, undergo changes. Zhang et al. developed a cross-linked network composed of alginate-dopamine (AD), chondroitin sulfate (CS), and regenerated silk fibroin (RSF) to create a highly adhesive hydrogel for wet surfaces (Zhang et al., 2021). When this hydrogel, encapsulating BMSC-Exos, was injected into rat knee joint cartilage defects, it promoted there cruitment of BMSCs by enhancing the action of BMSCs-Exos and facilitated the differentiation of BMSCs into chondrocytes. Xiaole Qi et al. have developed a multifunctional hydrogel delivery system that co-delivers MSC-Exos and Icariin (ICA) to harness their synergistic effects (Zeng et al., 2023). This mussel-inspired system utilizes a multifunctional hydrogel inspired by mussel adhesion, exhibiting thermal sensitivity, self-healing, and adhesive properties. These characteristics enhance the cellular uptake of MSC-Exos and ICA, prolonging their retention and demonstrating enhanced cartilage protection. Utilizing drug-loaded MSC-Exos can enhance their drug-loading efficiency through various strategies, including regulating the culture conditions of the parent cells and utilizing biomaterials to assist in delivery (Table 2).

**TABLE 2:** Engineering approaches to enhance drug delivery efficacy of MSC-Exos.

Strategy	Technology	Application in OA therapy	References
Precondition	Biological stimuli (like growth factor)	Enhances the production of MSC-Exos and the activity of anti-inflammatory and regenerative factors for OA symptom relief	Chen et al. (2022)
	Chemical stimuli (like small molecule)		
	Physical stimuli (like hypoxia, ultrasound)		Oses et al. (2017)
	Scaffolds		
Cargo loading	Co-incubation	Enables targeted delivery of pain relievers or regenerative agents to OA-affected joints	Zhang et al. (2023)
	Sonication/Electroporation/Extrusion		
	Chemical transfection/Reagent kit		
	Antibody binding		
Genetic edition	Retrovirus/Lentivirus/Baculovirus/Adenovirus/Adeno-associated virus/Plasmid transfection	Customizes MSC-Exos with tailored therapeutic molecules for OA symptom alleviation	Rahbaran et al. (2022)
	Electroporation		
	Liposomes and Polymers		
	Inorganic Nanoparticles		
Surface modification	Peptides modification	Facilitates site-specific drug delivery, ensuring precise targeting and enhanced efficacy	Liang et al. (2020)
	Chemical modification		
	Non-covalent modification		
Biomaterials assisted delivery	Natural/Synthetic polysaccharide hydrogels	Sustains the release of MSC-Exos within joints, supporting prolonged therapeutic effects	Leisheng et al. (2023)
	Biodegradable metal scaffolds		
	Bioactive ceramic biomaterials		Zhang et al. (2018a)
	Synthetic polymer materials		

## 6 Current studies of MSC-Exos in OA therapy

### 6.1 Potential of MSC-Exos therapeutics in OA

MSCs have been proven to possess the potential for differentiating into chondrocytes both *in vivo* and *in vitro*. As mentioned earlier, following OA cartilage injury, endogenous (from the pericellular matrix or circulating) or exogenous MSCs are recruited to the site of injury, where they partially undergo chondrogenic differentiation, facilitating cartilage regeneration. MSC-Exos influence MSC homing and promote chondrogenic differentiation. Yan Kang et al. utilized MSC-Exo delivery of circRNA_0001236 and found that it enhances MSC chondrogenesis through the miR-3677-3p/Sox9 axis, thereby suppressing cartilage degradation (Mao et al., 2021). However, the quantity of endogenous MSCs is limited, and the homing capacity of exogenous MSCs is restricted. Relying solely on MSC chondrogenesis yields suboptimal results. While MSC-Exos cannot directly increase the number of MSCs, they can enhance cartilage repair through alternative pathways. Tao Shu et al. employed miRNA-210-overexpressing BMSCs-Exos, significantly reducing LPS-induced cartilage injury via the NF-kβ pathway (He et al., 2020). AD-MSCs-Exos deliver miR-100-5p to chondrocytes, significantly enhancing cellular autophagy by inhibiting the mTOR pathway, consequently reducing cartilage damage. Animal experiments have demonstrated substantial gait improvements (Wu et al., 2019).

Inflammatory conditions within the joint cavity accelerate OA progression, often preceding radiological manifestations and significantly correlating with clinical pain. Thus, controlling early OA inflammation is crucial to slowing its progression and improving symptoms. MSCs modulate various immune cells within the joint. Researchers subjected AD-MSCs to inflammatory stimuli TNF-α and IFN-γ, finding increased expression of vascular endothelial growth factor and fibroblast growth factor genes, while pro-inflammatory factors TNFA and PTGS2 gene expression decreased, indicating that AD-MSCs can mitigate inflammation through paracrine effects in inflammatory environments (Sukho et al., 2017). After co-culturing AD-MSCs-Exos with IL-1β stimulated chondrocytes, AD-MSCs-Exos reduced the activity of inflammatory transcription factors, thereby decreasing the expression of inflammatory mediators, exerting a protective effect on the chondrocyte extracellular matrix. Shen et al. (2016) discovered that MSC-Exos express C-C motif chemokine receptor 2 (CCR2), acting as decoys that bind and inhibit the activity of the pro-inflammatory chemokine CCL2, preventing macrophage accumulation and tissue damage. Moreover, MSC-Exos target macrophages, regulating M1/M2 polarization, a phenomenon demonstrated in various disease models. MSC-Exos inhibit T cell proliferation in a dose-dependent manner and induce Treg populations, thereby exerting immunomodulatory effects on inflammatory joint diseases.

**FIGURE 2:**
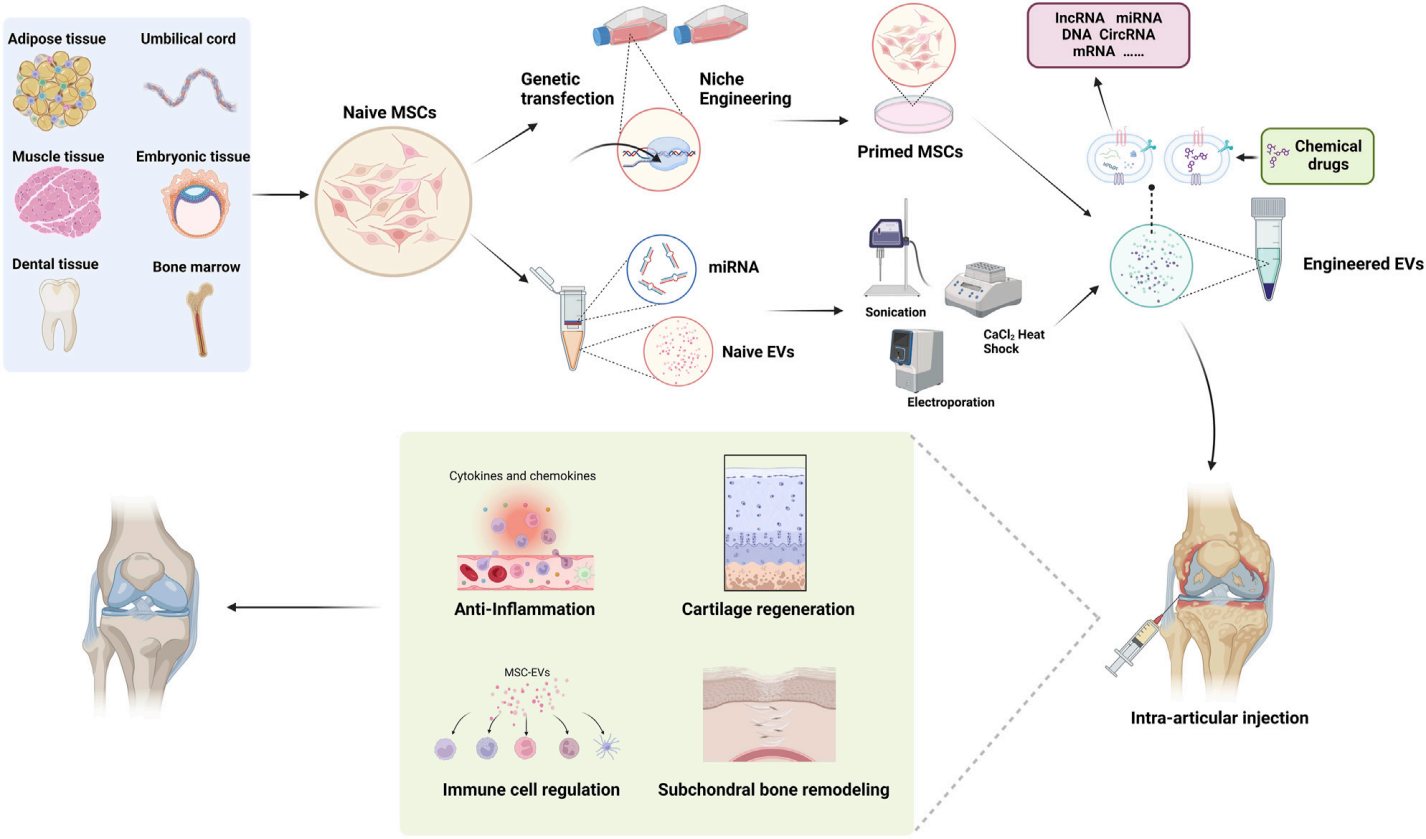
Schematic images of MSC-Exos as drug carrier for OA treatment.

Michelle Delco et al. discovered that MSCs package fully functional mitochondria into exosomes, which are then transferred to chondrocytes. This restoration of mitochondrial function in recipient cells maintains cellular vitality, promoting tissue repair (Fahey et al., 2022). This work provides a delivery strategy for cell-free mitochondrial targeted therapy, highlighting the potent therapeutic potential of mitochondrial delivery in poorly vascularized musculoskeletal tissues. Given the excellent delivery capabilities and rich biological functionalities of MSC-Exosomes, their potential as a drug delivery system for treating OA is remarkable, with research in relevant models becoming increasingly mature (Figure 2).

### 6.2 Application of MSC-Exos based drug delivery systems in OA treatment

**FIGURE 3:**
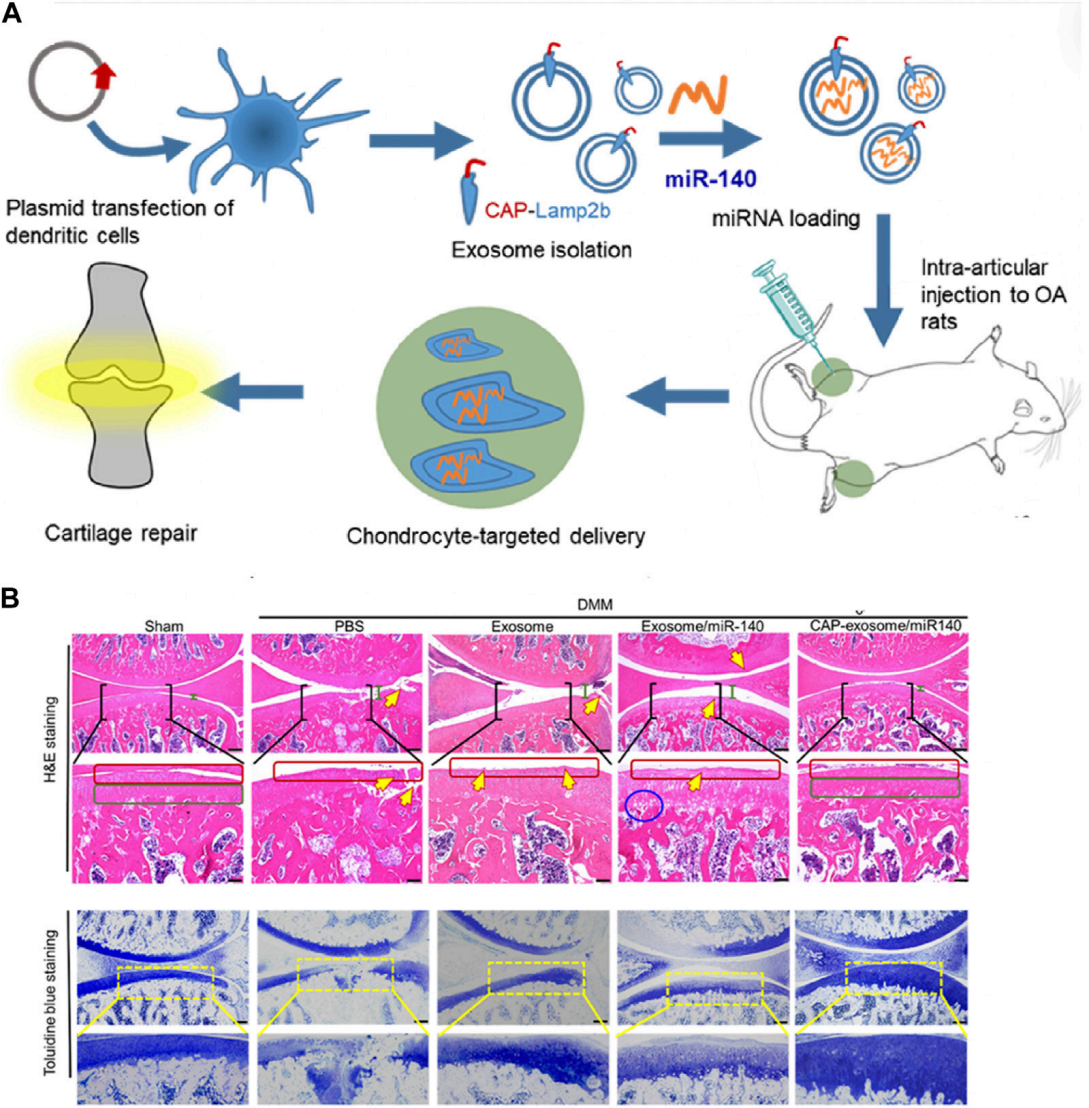
Representative study on MSC-Exos drug delivery for OA therapy. **(A)** Schematic illustration of surface engineering of exosomes for targeted delivery of miR-140 to chondrocytes for OA treatment. **(B)** Histological assessment of the cartilage of DMM rats treated with CAP-exosome/miR-140 or exosome/miR-140 preparations: Stained images of the cartilage tissues. Scale bars, 250 μm for the top panel and 100 μm for the bottom panel. Certain regions of the top panel pictures are enlarged in the bottom panel. Green bars indicate the distance of the joint space. Yellow arrows indicate fractures or irregular morphology of the cartilage layer. Red squares indicate the surface of the cartilage layers. The blue circle indicates cavities between cartilage and subchondral bone. Green squares indicate normal, condensed morphology of the cartilage. Reprinted (adapted) with permission from (Liang et al., 2020). Copyright {2024}American Chemical Society.

Research on cell-free therapy using MSC-Exos lags behind MSC therapy. Currently, the development of Targeted Drug Delivery Systems (DDS) based on MSC-Exos is in its early stages, primarily limited to *in vitro* or animal experiments. We have summarized representative studies from the past 5 years (Table3). The most frequently applied MSC-Exo delivery involves various microRNAs. Various microRNAs have been proven to be deliverable to chondrocytes or immune cells within the joints, improving OA prognosis through promoting chondrogenesis, inhibiting apoptosis, and immune modulation. A single microRNA can have multiple potential targets. For instance, studies conducted by Wang R et al. have demonstrated that microRNA-135p, when loaded onto MSC-Exos for treating OA, targets genes such as PDGF-BB, MAPK6, and Sp1, influencing the biological functions of various components in joint cartilage, subchondral bone, and immune cells, thereby enhancing OA prognosis (Wang and Xu, 2021; Wang and Xu, 2022).Liang et al. utilized CAP-labeled exosomes, characterized by prolonged joint retention, limited *in vivo* diffusion, and the ability to deliver miR-140 to the deep cartilage region. This delivery effectively suppressed cartilage-degrading enzymes, thereby alleviating the progression of osteoarthritis in a rat model (Figure 3).

**TABLE 3:** Representative studies on MSC-Exos drug delivery for OA therapy in last 5 years.

Sources	Isolation	Model	Cargo loading	Target	Biological effect	References
AD-MSCs	UC	Rats (MIA)	miR-376c-3p	WNT3	Mitigate chondrocyte degradation and synovial fibrosis	Li et al. (2023)
			Natural cargo	WNT9a		
SMSCs	Hieff^TM^ EEK	Rats (DMM)	miR-320c	ADAM19	Suppresses ECM degradation and chondrocyte apoptosis	Kong et al. (2023)
			Lipofectamine Trans			
BMSCs	UC	Mice chon-drocyte (COL II)	lncRNA KLF3-AS1	YBX1	Inhibit IL-1 β induced autophagy and apoptosis of chondrocytes through activating the PI3K/Akt/mTOR pathway	Wen et al. (2022)
			Plasmid Trans			
BMSCs	UC	Mice (DMM)	circRNA_0001236	miR-3677-3p	Regulate cartilage metabolic balance and promote chondrogenic differentiation	Mao et al. (2021)
			Plasmid Trans			
SF-MSCs	UC	Rats (ACLT)	Kartogenin	—	Enable *in situ* chondrogenic differentiation of the transplanted SF-MSCs for cartilage regeneration	Xu et al. (2021)
			Electroporation			
BMSCs	UC	Rats chon-drocyte (IL-1β)	miR-127-3p	CDH11	Blocking the Wnt/β-catenin pathway activation to relieve chondrocyte damage	Dong et al. (2021)
			Natural cargo			
SMSCs	UC	OA PHCs	miR-129-5p	HMGB1	Declined the inflammatory response and apoptosis of chondrocytes	Qiu et al. (2021)
			Lipofectamine Trans			
BMSCs	UC	Rats (ACLT)	miR-361-5p	DDX20	Alleviates OA damage by inactivating the NF-κβ signaling pathway	Tao et al. (2021)
			Lipofectamine Trans			
BMSCs	UC	Rats (ACLT)	miR-9-5p	SDC1	Reduce levels of inflammatory factors and oxidative stress damage	Jin et al. (2020)
			Lipofectamine Trans			
BMSCs	UC	OA PHCs	miR-136-5p	ELF3	Promotes chondrocyte proliferation and inhibits chondrocyte degeneration	Chen et al. (2020)
			Oligonucleotide Trans			
BMSCs	UC	Mice	Curcumin	miR-143	Reduce chondrocytes apoptosis	Qiu et al. (2020)
			Co-culture	miR-124		
hIPFP-MSCs	ExoQuick ™ EEK; UC	Mice (DMM)	miR-100-5p	mTOR	Maintaining cartilage homeostasis	Wu et al. (2019)
AD-MSCs			Natural cargo			
BMSCs	UC	OA PHCs	miR-92a-3p	WNT5A	Promotes chondrocytes migration, proliferation and differentiation	Mao et al. (2021)
			Lipofectamine Trans			
BMSCs	Thermo Scientific^TM^ EEK	Rats (COL II)	lncRNA KLF3-AS1	—	Suppressed IL-1β-induced apoptosis of chondrocytes, promoted cartilage repair and chondrocyte proliferation	Liu et al. (2018)
			Lentivirus Trans			
BMSCs	UC	Mice (ACLT)	miR-135b	PDGF-BB	Inhibits abnormal angiogenesis in subchondral bone and alleviate OA-induced pain and bone resorption	Wang and Xu (2022)
			TGF-β stimulated	MAPK6	Promoted M2 polarization of synovial macrophages	Wang and Xu (2021)
	Genese-ed^TM^ EEK	Rats (DMM)		Sp1	Promoted chondrocyte proliferation	Wang et al. (2018)

Abbreviations: UC, Ultracentrifugation; EEK, exosome extraction kit; OA PHCs, osteoarthritis primary human chondrocytes; MIA, monosodium iodoacetate; ACLT, anterior cruciate ligament transaction; DMM, destabilization of the medial meniscus; COL II, collagenase II induced; Trans, Transfection; Abbreviations for genes and proteins can be found at the end of the paper.

Animal models of OA are categorized as spontaneous and induced. Spontaneous models include natural occurrence and genetic models. However, due to their lengthy experimental periods, poor controllability, and high costs, they are less commonly utilized. Transgenic mouse OA models, studying the effect of individual genes in the pathogenesis of OA, have yielded good results. Nevertheless, OA is generally believed to result from the interaction of multiple genes, making such models oversimplified in representing the development of OA. Constructing animal models of OA typically involves two main approaches: intra-articular drug injections and joint surgery. Surgical induction, mainly performed on the knee joint, includes procedures like partial or complete meniscectomy, anterior cruciate ligament transection, and medial or lateral collateral ligament transection. The goal is to induce joint instability and mechanical structure changes, making it the mainstream modeling method due to its short modeling period and clear effects (Liu et al., 2016). However, it is considered closer to the development process of post-traumatic osteoarthritis. Chemical induction primarily employs sodium iodate and collagenase. While this method yields rapid results, its effectiveness as a clinical model is questioned, often being used more as a joint pain model (Barton et al., 2017).

As of September 2023, there are 238 registered clinical trials for MSC therapy for OA listed on the National Institutes of Health (NIH) database. The safety and effectiveness of MSC therapy have been largely validated. However, there is only one registered clinical trial related to MSC-Exos (NCT05060107), initiated by Espinoza F in October 2021. The study aims to evaluate the safety of exosomes from allogeneic mesenchymal stromal cells delivered via intra-articular injection in the knee of patients with mild to moderate symptomatic osteoarthritis. They plan to enroll 10 patients in this phase 1 trial with a follow-up period of up to 12 months. However, the experimental results have not been published yet. Generating clinical-grade MSC-exos for OA treatment faces hurdles including standardization of isolation techniques, scalability of production, and ensuring safety and efficacy. Ensuring consistency in yield, purity, and biological activity is vital, as is rigorous quality control for safety and efficacy. Optimizing therapeutic efficacy and navigating regulatory requirements also present challenges (Harrell et al., 2020). It is believed that there will be more clinical trials in the future to validate the effectiveness of MSC-Exos in drug delivery for OA. Upgrading exosome production techniques to reduce economic costs, combined with new biomaterials, opens new avenues for drug delivery strategies. This integration is crucial for the clinical application of MSC-Exos.

## 7 Conclusion and outlook

The current non-surgical treatments for osteoarthritis are not very effective due to the irreparable damage to cartilage, posing a significant challenge. With the advancements in regenerative medicine, cell therapies, particularly those involving MSCs, have been extensively researched. These studies have shown promising results in various preclinical trials. The use of MSC-Exos to treat osteoarthritis not only overcomes ethical limitations associated with cell therapies but also harnesses their potential as drug carriers. One notable advantage of exosomes is their editability, which plays a crucial role in enhancing their efficacy as drug carriers. Collaboration among researchers from different fields, including molecular biology, nanotechnology, and material science, is vital in developing exosome-based drug delivery systems with stronger targeting capabilities and higher drug-loading efficiency. As efforts focus on improving drug-loading efficiency, future research will also address the need for sustained drug release within the joint cavity. This approach aims to significantly reduce the frequency of intra-articular injections, thereby lowering economic costs and minimizing side effects.
